# Supplementary material for: Cytokine Expression and Haptoglobin Levels in Bovine Fetuses Spontaneously Aborted by Intracellular Infectious Agents and by Probable Infectious Etiology
Source: Animals (Basel). 2025 Oct 1;15(19):2878. doi: 10.3390/ani15192878 (PMC12523439; doi:10.3390/ani15192878)
Supplement: Supplementary file 1 [file animals-15-02878-s001.zip › animals-3868127-supplementary.pdf]

Table S1. Information on selected bovine aborted fetuses due to “intracellular infectious agents” and “probable infectious etiology”, as well as control fetuses. The table includes data on the production system, sex, gestational age, and the most relevant macroscopic and microscopic lesions observed in fetal tissues.

**Group**	**Infectious agent**	**Number**	**Gestational age (months)**	**Production system**	**Sex**	**Autolysis (Score)**	**Main lesions Gross**	**Histopathology**
Intracellular agent	*N. caninum*	1	4	Dairy	Male	2	NL	necrotizing meningoencephalitis, epicarditis, myocarditis, necrotizing hepatitis, myositis, glossitis, intestinal serositis and interstitial pneumonia
		2	5	Dairy	Male	2	Hepatomegaly	necrotizing meningoencephalitis, epicarditis, myocarditis, portal hepatitis, necrotizing myositis, glossitis, interstitial nephritis and interstitial pneumonia
		3	5	Beef	Female	1	NL	necrotizing meningoencephalitis, epicarditis, necrotizing myocarditis, endocarditis, glossitis, interstitial nephritis and placentitis
		4	4	Beef	Male	2	NL	necrotizing meningoencephalitis, epicarditis (Fig. 1A), myocarditis, portal hepatitis, interstitial nephritis, myositis, enteritis, intestinal serositis, interstitial pneumonia and pleuritis
		5	8	Beef	Male	1	Reticular pattern on liver surface	necrotizing meningoencephalitis, epicarditis, myocarditis and interstitial pneumonia
		6	7	Beef	Female	1	NL	meningoencephalitis and interstitial pneumonia
		7	7	Dairy	Female	1	Hepatomegaly<br>Reticular pattern on liver surface<br>Lymphadenomegaly	meningoencephalitis, epicarditis, myocarditis, endocarditis, multifocal and portal hepatitis, hepatic serositis and interstitial pneumonia
		8	Full term	Beef	Female	1	NL	encephalitis, epicarditis, myocarditis and portal hepatitis
		9	Full term	Dairy	Male	2	Reticular-looking surface in liver	necrotizing encephalitis (Fig. 1B), epicarditis, myocarditis and portal hepatitis
	B. abortus	10	5	Beef	Male	1	Hepatomegaly	bronchopneumonia, epicarditis, portal hepatitis, enteritis and placentitis
		11	Full term	Beef	Female	1	NL	bronchopneumonia (Fig. 1C), interstitial pneumonia, pleuritis and epicarditis
	BVDV	12	5	Dairy	Male	1	NL	myocarditis, portal hepatitis (Fig. 1D) and enteritis

		13	7	Dairy	Female	1	NL	encephalitis and myositis
Possible infectious abortion	-	14	8	Beef	Female	1	NL	epicarditis, myocarditis, glossitis and interstitial pneumonia
	-	15	Full term	Beef	Male	1	Hepatomegaly Lymphadenomegaly	epicarditis, myocarditis and interstitial pneumonia
	-	16	Full term	Beef	Male	1	NL	encephalitis and portal hepatitis
	-	17	Full term	Beef	Female	1	Hepatomegaly	encephalitis, myocarditis, portal hepatitis, myositis, glossitis and enteritis (Fig. 1E)
	-	18	Full term	Beef	Female	2	Hepatomegaly	multifocal pneumonia (Fig. 1F)
Control fetuses	-	19	4	Beef	Female	0	NL	NL
	-	20	5	Beef	Female	0	NL	NL
	-	21	5	Beef	Male	0	NL	NL
	-	22	Full term	Beef	Female	0	NL	NL
	-	23	Full term	Beef	Male	0	NL	NL
	-	24	Full term	Beef	Female	0	NL	NL
	-	25	Full term	Beef	Male	0	NL	NL
	-	26	Full term	Beef	Male	0	NL	NL
	-	27	Full term	Beef	Female	0	NL	NL
	-	28	8	Beef	Male	0	NL	NL

NL: no gross and histopathology lesion observed.

Table S2. Ct values for each gene corresponding to each sampled fetus used for statistical analysis in REST (Qiagen Inc., Valencia, CA, USA).

Sample Protocol n°	Infection	Age months	GAPDH	IFNγ	TNFα	IL-4	IL-8	IL-12
1	*N. caninum*	4	29,02	29,12	-	-	-	-
2		4	22,04	24,47	25,14	-	25,56	34,46
3		5	26,86	31,27	34,5	-	34,11	-
4		5	23,48	28,75	-	-	29,67	-
5		7	23,48	28,75	-	-	29,67	31,94
6		7	25,64	26,64	-	-	32,11	35,07
7		8	22,04	24,47	25,14	-	25,56	34,46
8		Full term	26,86	31,27	-	-	34,11	-
9		Full term	23,48	28,75	34,5	30,34	29,67	31,94
10	*B. abortus*	5	24,66	29,36	29,19	-	30,51	36,67
11		Full term	24,79	29,26	25,77	-	29,95	34,03
12	BVDV	5	22,04	26,75	28,91	29,7	27,2	34,92
13		7	28,95	25,53	33,21	-	37,01	-
14	PIE	8	30,41	27,15	31,05	-	28,89	33,61
15		Full term	26,04	33,31	29,08	-	29,27	35,31
16		Full term	26,69	26,91	34,4	-	31,09	36,08
17		Full term	26,06	30,55	33,9	34,92	27,87	-
18		Full term	23,08	26,47	-	31,47	25,71	32,84
19	Control fetuses	4	21,37	30,4	30,64	-	32,45	-
20		5	21,16	29,36	25,59	-	29,58	35,32
21		5	32,44	-	-	-	-	-
22		8	22,62	25,75	26,73	-	-	31,94
23		Full term	27,29	26,68	30,93	34,87	31,18	-
24		Full term	28,63	28,84	27,6	34,61	27,85	34,86
25		Full term	30,76	25,3	28,91	31,55	37,29	-
26		Full term	27,02	26,73	26,88	-	28,77	36,07
27		Full term	24,06	23,99	30,85	-	27,21	34,3

28	Full term	26,74	-	27,98	-	24,01	35,68

Report on the average Ct for the two technical replicates corresponding to each fetal sample. These were used for statistical analysis using REST (Qiagen Inc., Valencia, CA, USA). PIE: probable infectious etiology.

Table S3. Relative expression analysis of target genes performed with REST (Qiagen Inc., Valencia, CA, USA) in the spleens of naturally aborted bovine fetuses due to intracellular agents in mid-gestation (MGF) and late gestation (LGF).

Gene	Treatment	change	Max. error	Min. error	p-value	Signif.
	Control MGF	1				
IFNγ	Intracellular MGF	10,660	20,097	4,943	0,040	UP
(1)	Control LGF	1				
	Intracellular LGF	0,049	0,288	0,006	0,012	DOWN
	Control MGF	1				
TNFα	Intracellular MGF	2,238	24,300	0,269	0,540	-
(1)	Control LGF	1				
	Intracellular LGF	0,456	6,355	0,045	0,502	-
	Control MGF	1				
IL-8	Intracellular MGF	9,429	17,932	2,949	0,000	UP
(1)	Control LGF	1				
	Intracellular LGF	0,063	0,983	0,006	0,011	DOWN
	Control MGF	1				
IL-12	Intracellular MGF	1,698	5,583	2,834		-
(0,773)	Control LGF	1				
	Intracellular LGF	0,472	1,505	0,123	0,372	-
	Control MGF	1				
IL-4	Intracellular MGF	-	-	-		-
(0,805)	Control LGF	1				
	Intracellular LGF	2,038	4,528	1,626	0,922	-

Randomization tests were carried out with 2000 iterations (default setting, [40]). GAPDH was used as the reference gene, based on its widespread application in bovine studies and confirmed stability in the present dataset. The efficiency of each gene is expressed in parentheses below the gene; for GAPDH, the efficiency was 0.913. Exact p-values generated by REST are provided for each comparison, together with fold change and confidence intervals.

Table S4. Relative expression analysis of target genes performed with REST (Qiagen Inc., Valencia, CA, USA) in the spleens of naturally aborted bovine fetuses due to probable infectious causes (PAI).

Gene	Treatment	change	Max. error	Min. error	p-value	Signif.
IFNγ	Control	1				
	PAI	0,017	0,617	0,005	0,000	DOWN
TNFα	Control	1				
	PAI	0,123	1,722	0,007	0,116	-
IL-8	Control	1				
	PAI	0,383	9,795	0,024	0,503	-
IL-12	Control	1				
	PAI	1,480	7,324	0,276	0,833	-
IL-4	Control	1				
	PAI	0,213	0,544	0,049	0,220	-

Randomization tests were carried out with 2000 iterations (default setting, [40]). GAPDH was used as the reference gene, based on its widespread application in bovine studies and confirmed stability in the present dataset. Exact p-values generated by REST are provided for each comparison, together with fold change and confidence intervals.

Table S5. Relative expression analysis of target genes performed with REST (Qiagen Inc., Valencia, CA, USA) in the spleens of naturally aborted bovine fetuses due to intracellular agents and probable infectious causes (PAI).

Gene	Treatment	change	Max. error	Min. error	p-value	Signif.
IFNγ	PAI	1				
	Intracellular	1,815	5,047	0,520	0,412	-
TNFα	PAI	1				
	Intracellular	1,732	16,211	0,136	0,698	-
IL-8	PAI	1				
	Intracellular	0,070	0,190	0,041	0,010	DOWN
IL-12	PAI	1				
	Intracellular	0,653	1,893	0,321	0,761	-
IL-4	PAI	1				
	Intracellular	12,338	16,743	8,886	0,000	UP

Randomization tests were carried out with 2000 iterations (default setting, [40]). GAPDH was used as the reference gene, based on its widespread application in bovine studies and confirmed stability in the present dataset. Exact p-values generated by REST are provided for each comparison, together with fold change and confidence intervals.
